# Nutrition and Training Influences on the Regulation of Mitochondrial Adenosine Diphosphate Sensitivity and Bioenergetics

**DOI:** 10.1007/s40279-017-0693-3

**Published:** 2017-03-22

**Authors:** Graham P. Holloway

**Affiliations:** Department of Human Health and Nutritional Sciences, University of Guelph, 491 Gordon St., Guelph, ON N1G 2W1 Canada

## Abstract

Since the seminal finding almost 50 years ago that exercise training increases mitochondrial content in skeletal muscle, a considerable amount of research has been dedicated to elucidate the mechanisms inducing mitochondrial biogenesis. The discovery of peroxisome proliferator-activated receptor γ co-activator 1α as a major regulator of exercise-induced gene transcription was instrumental in beginning to understand the signals regulating this process. However, almost two decades after its discovery, our understanding of the signals inducing mitochondrial biogenesis remain poorly defined, limiting our insights into possible novel training modalities in elite athletes that can increase the oxidative potential of muscle. In particular, the role of mitochondrial reactive oxygen species has received very little attention; however, several lifestyle interventions associated with an increase in mitochondrial reactive oxygen species coincide with the induction of mitochondrial biogenesis. Furthermore, the diminishing returns of exercise training are associated with reductions in exercise-induced, mitochondrial-derived reactive oxygen species. Therefore, research focused on altering redox signaling in elite athletes may prove to be effective at inducing mitochondrial biogenesis and augmenting training regimes. In the context of exercise performance, the biological effect of increasing mitochondrial content is an attenuated rise in free cytosolic adenosine diphosphate (ADP), and subsequently decreased carbohydrate flux at a given power output. Recent evidence has shown that mitochondrial ADP sensitivity is a regulated process influenced by nutritional interventions, acute exercise, and exercise training. This knowledge raises the potential to improve mitochondrial bioenergetics in the absence of changes in mitochondrial content. Elucidating the mechanisms influencing the acute regulation of mitochondrial ADP sensitivity could have performance benefits in athletes, especially as these individuals display high levels of mitochondria, and therefore are subjects in whom it is notoriously difficult to further induce mitochondrial adaptations. In addition to changes in ADP sensitivity, an increase in mitochondrial coupling would have a similar bioenergetic response, namely a reduction in free cytosolic ADP. While classically the stoichiometry of the electron transport chain has been considered rigid, recent evidence suggests that sodium nitrate can improve the efficiency of this process, creating the potential for dietary sources of nitrate (e.g., beetroot juice) to display similar improvements in exercise performance. The current review focuses on these processes, while also discussing the biological relevance in the context of exercise performance.

## Introduction

Strenuous exercise can increase the energetic demands of skeletal muscle by 100-fold over resting requirements, placing an enormous challenge on bioenergetic pathways to maintain concentrations of adenosine triphosphate (ATP), the basic unit of energy within muscle. Skeletal muscle is equipped with an intricate series of enzymatic reactions that resynthesize ATP to ensure cellular survival during these conditions. The diverse reactions buffering ATP fluctuations within muscle display a continuum of speed (i.e., maximal rate of ATP production) relative to the capacity for ATP production. For instance, the breakdown of phosphocreatine (PCr) can produce ATP extremely rapidly for a relatively short period of time, while in contrast, aerobic metabolism within mitochondria produces ATP at a lower maximal rate, but essentially has a limitless capacity. As a result, aerobic metabolism dominates ATP production during most continuous exercise situations, highlighting the importance of mitochondria to overall metabolic homeostasis. This simplistic view of metabolism is efficient for teaching purposes as it enables ‘compartmentalization’ of each system; however, in reality, there is considerable integration between each bioenergetic process. Historically, an increase in mitochondrial content represents a fundamental exercise training response [1–4], contributing to the improvement in maximal aerobic capacity and the improvement in submaximal exercise performance, a process largely influenced by altering glycolytic flux (i.e., glycogen breakdown). This review focuses on discussing (1) the biological influence of increasing mitochondrial content and (2) signals regulating mitochondrial biogenesis. The biological consequence of increasing mitochondrial content is an improvement in adenosine diphosphate (ADP) sensitivity (as discussed in Sect. 4), and recent evidence has suggested that this process is externally regulated. Therefore, this review also discusses (3) nutritional and training strategies to augment mitochondrial bioenergetics in the absence of the induction of mitochondrial biogenesis.

## Historical Perspective

It has been known for almost a century that elite athletes have a higher maximal rate of oxygen consumption, higher maximal mitochondrial enzymatic activities, and ultimately improved performance. While originally attributed to genetics, Holloszy’s landmark research in 1967 demonstrated the remarkable plasticity of skeletal muscle to increase mitochondrial content and improve exercise capacity [2]. This seminal paper described the basic observation that overload training increases mitochondrial content, but does not alter the intrinsic function of mitochondria. As a result, research in the subsequent 50 years has focused on understanding the biological relevance of altering mitochondrial content while also elucidating the mechanisms responsible for the induction of mitochondrial biogenesis.

**Fig. 1:**
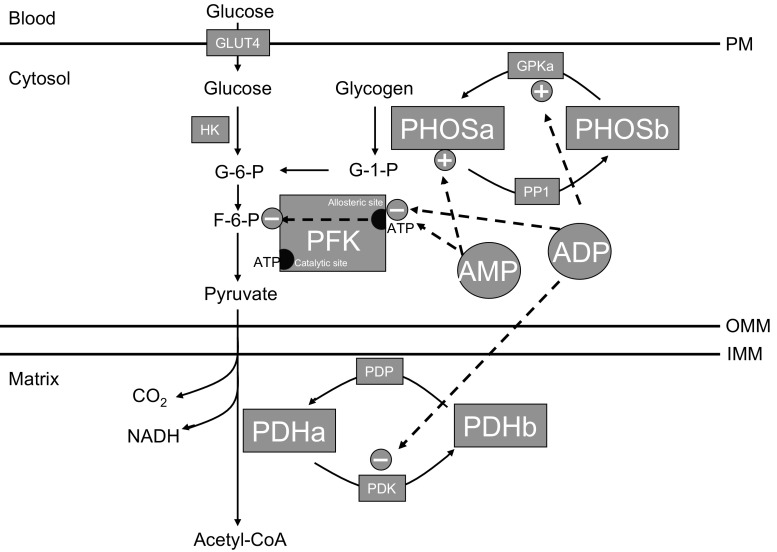
Importance of adenosine diphosphate (ADP) and adenosine monophosphate (AMP) in the regulation of metabolism. This schematic overview highlights the importance of ADP and AMP in the regulation of key enzymes in carbohydrate metabolism, specifically glycogen phosphorylase (PHOS), phosphofructokinase (PFK), and pyruvate dehydrogenase (PDH). Covalent regulation exists for PHOS and PDH, as the phosphorylation status renders these enzymes active (a form) and less active (b form) forms of the enzymes. *ATP* adenosine triphosphate, *G-1-P* glucose-1-phosphate, *G-6-P* glucose-6-phosphate, *F-6-P* fructose-6-phosphate, *GLUT* glucose transporter, *GPK* glycogen phosphorylase kinase, *HK* hexokinase, *IMM* inner mitochondrial membrane, *NADH* nicotinamide adenine dinucleotide, *OMM* outer mitochondrial membrane, *PM* plasma membrane, *PP1* glycogen pyrophosphatase, *PDK* pyruvate dehydrogenase kinase, *PDP* pyruvate dehydrogenase phosphatase

In 1984, Holloszy postulated that an increase in mitochondrial content would alter the sensitivity of oxidative metabolism to ADP [4], suggesting less ADP would be required to stimulate a given amount of aerobic ATP production. This basic working model has been supported several ways. Originally, Dudley and colleagues used rodent skeletal muscles to illustrate a relationship between free ADP levels and mitochondrial content during muscle contraction [5], and, in humans, training has repeatedly been shown to attenuate the typical rise in free ADP during exercise [6, 7]. While several processes could influence free ADP concentrations in vivo (e.g., increased force per ATP), direct assessments of mitochondrial respiration using permeabilized muscle fibers have consistently shown an improvement in respiration at a submaximal ADP level following training [8, 9], or conversely a reduction in the amount of ADP required to maintain a given aerobic flux, suggesting mitochondrial changes contribute to the improvement in ADP sensitivity following training. The reduction in free ADP is a key molecular event contributing to training-induced improvements in exercise performance, as this spares glycogen utilization at any power output, enabling an athlete to work at a higher power output with the same ‘metabolic stress’ to the muscle. While most enzymes in metabolic pathways are near equilibrium, there are a few reactions within the glycolytic system that are considered ‘rate limiting’, and are externally regulated. These enzymes include glycogen phosphorylase, phosphofructokinase, and pyruvate dehydrogenase (PDH), and ADP, or the subsequent accumulation of adenosine monophosphate (AMP), stimulate flux through all three of these enzymes (Fig. 1).

Specifically, ADP activates glycogen phosphorylase kinase to covalently modify phosphorylase, while AMP allosterically regulates phosphorylase by increasing the sensitivity of the enzyme to glycogen, promoting glycogen breakdown. In the context of phosphofructokinase, ADP and AMP decrease the sensitivity of the allosteric inhibitory binding site for ATP, essentially removing an inhibitor for enzymatic flux, while ADP also inhibits PDH kinase activity, maintaining PDH in an active state and promoting pyruvate flux towards aerobic respiration. As a result, a reduction in cytosolic ADP levels, and the subsequent production of AMP, as occurs following training [7], provides less of a metabolic stimulus for glycogen utilization, shifting fuel preference towards fatty acids, and improves exercise tolerance. An increase in mitochondrial content is therefore thought to represent a key event influencing training adaptations, as the ensuing improvement in ADP sensitivity during exercise, attenuates the rise in cytosolic ADP and decreases glycogen utilization.

## Mitochondrial Biogenesis

Given that increasing mitochondrial content represents a key mechanism for the improvement in exercise performance following training, it is understandable that research has focused on elucidating the molecular events responsible for the initiation of mitochondrial biogenesis. The induction of mitochondrial biogenesis requires the transcription of proteins contained within both the nuclear and the mitochondrial DNA genomes. The mitochondrial proteome consists of ~1600 proteins, the vast majority of which are encoded within the nucleus, as the mitochondrial DNA only encodes for 13 subunits of the electron transport chain and proteins required for messenger RNA translation within this organelle (for a review, see Hood [10]). The induction of mitochondrial biogenesis therefore involves a coordinated signaling response that stimulates both genomes. The identification of peroxisome proliferator-activated receptor γ co-activator 1α (PGC-1α) as a transcriptional co-activator synchronizing this process was a major advancement in our understanding of the molecular mechanisms regulating mitochondrial content [11]. Since the discovery of PGC-1α, the complexity of the signals regulating mitochondrial biogenesis continues to be expanded, as splice variants of PGC-1α have been identified [12], the subcellular location of PGC-1α is acutely regulated [13, 14], and it has been shown that PGC-1α is phosphorylated and acetylated [15–17]. While research continues to develop our understanding of the processes involved in expanding the mitochondrial volume (for a detailed review, see Joseph and Hood [18]), our knowledge of the signals initiating mitochondrial biogenesis remains poorly defined.

A large body of literature has been devoted to studying the role of energy turnover and activation of AMP activated protein kinase (AMPK) as a key signal to induce mitochondrial biogenesis. However, high-intensity interval training and ‘classical’ endurance training result in similar accumulations of mitochondrial proteins, suggesting this process is not directly influenced by the rate of ATP hydrolysis [19]. In addition, genetic models that render AMPK activity substantially impaired have been commonly employed to determine the role of AMPK activation in the induction of mitochondrial biogenesis. Most genetic models have been confounded by potential impairments in cardiovascular performance during exercise [20, 21], making interpretations difficult. However, a recent model used skeletal muscle-specific ablation of liver kinase B1, an upstream activator of AMPK, to illustrate the impact of almost complete inactivation of AMPK during exercise [22]. While these mice display impaired exercise capacity and reduced mitochondrial content in sedentary animals, muscle-specific liver kinase B1 null mice responded normally to exercise training [22], suggesting activation of AMPK is not required for the induction of mitochondrial biogenesis.

The other ‘signals’ implicated in the regulation of mitochondrial content include calcium [specifically Ca^2+^/calmodulin-dependent protein kinase (CaMKII)] and reactive oxygen species (ROS)-mediated processes. Activation of CaMKII has been shown to induce the nuclear translocation of PGC-1α and the expression of mitochondrial genes [14], while pharmacological inhibition of this signaling pathway prevents exercise-mediated transcriptional responses [14]. While ROS has been considered a signal to induce mitochondrial biogenesis, clear evidence for a mechanistic role for ROS until recently has not been established, and instead a theoretical argument has largely been made based on observations that exercise increases oxidative damage of muscle [23]. However, several lines of evidence have now been established to implicate ROS, and specifically mitochondrial-derived ROS, in the induction of mitochondrial biogenesis. First, consumption of a high-fat diet has been shown to increase mitochondrial content [24, 25], a response associated with increased mitochondrial ROS, redox alterations of the calcium release channel (RyR), and activation of calcium signaling (CaMKII) [26]. Moreover, the consumption of a mitochondrial-targeted antioxidant [the Skulachev ion (SkQ)] prevented high-fat diet-induced mitochondrial biogenesis in association with unaltered CaMKII activation [26]. These data suggest overlap between ROS and calcium signaling, and have implicated mitochondrial ROS as a key signal in the initiation of mitochondrial biogenesis. While these data are focused on nutritional responses as opposed to exercise, the cell does necessarily differentiate between a high-fat diet and exercise, but rather adapts to a cellular stress to maintain homeostasis, in this case, redox stress, through the induction of mitochondrial biogenesis. In support of this concept, a recent paper has also outlined similar signaling events following exercise. Specifically, Place et al. [27] reported that a single bout of high-intensity interval training increased ROS-mediated fragmentation of the RyR in association with the induction of mitochondrial biogenesis. Moreover, these authors showed that mitochondrial ROS emission was attenuated after chronic training [27], potentially accounting for the diminished returns of exercise training with respect to continued expansion in mitochondrial volume. Altogether, these data suggest that mitochondrial-derived ROS is a key molecular signal for the induction of mitochondrial biogenesis, a process that may require redox modifications of the RyR and calcium-mediated signaling.

The notion that mitochondrial ROS contributes to exercise-induced mitochondrial biogenesis may help explain the lack of mitochondrial biogenesis observed in humans consuming antioxidants while exercise training [28]. In this manner, increasing the redox stress during exercise training may increase mitochondrial biogenesis. While one option would be to administer pro-oxidative compounds before training to achieve this, caution would be warranted with this approach as oxidative damage is known to compromise muscle performance [29, 30] and could also lead to mechanisms associated with overtraining (low-frequency fatigue). Alternatively, repeated bouts of exercise would be expected to result in a similar response, as the initial training bout would diminish the antioxidant capacity (e.g., reduced glutathione) within muscle, making it easier to induce ROS signaling in subsequent bouts of exercise. This may explain the previous work from John Hawley’s group, which has shown two bouts of exercise a day increases mitochondrial content in already trained individuals, while similar work separated over consecutive days does not [31]. While the authors have attributed the responses observed to training in a low-carbohydrate environment and have attempted to link the induction of mitochondrial biogenesis to AMPK signaling [31], given the recent data linking ROS to RyR modifications discussed above, an alternative explanation could relate to redox signaling. Clearly, future research is required to fully delineate the role of redox signaling in the induction of mitochondrial biogenesis, and to establish novel training paradigms to maximize these processes in athletes.

## Mitochondrial ADP Sensitivity

The induction of mitochondrial biogenesis, and the subsequent improvement in mitochondrial ADP sensitivity, has become synonymous with exercise training adaptations. This classical working model is premised on the belief that mitochondrial ‘function’ remains unaltered following a chronic training intervention. However, evidence is starting to accumulate to suggest that mitochondrial ADP transport is a regulated process, raising the possibility that lifestyle interventions can influence mitochondrial ADP sensitivity in the absence of the induction in mitochondrial biogenesis.

**Fig. 2:**
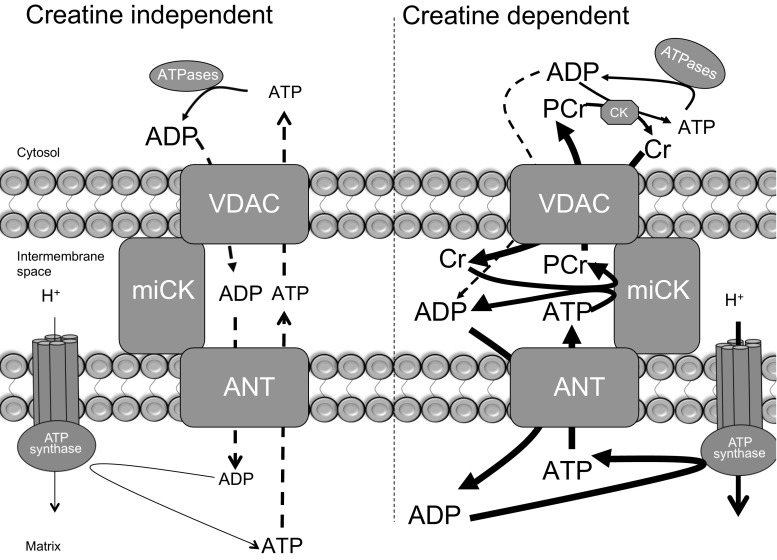
Schematic of adenosine diphosphate (ADP) and adenosine triphosphate (ATP) shuttling mechanisms of energy transfer across mitochondrial membranes. In both the presence and absence of creatine (Cr), adenine nucleotide transfer is believed to occur by diffusion through a voltage-dependent anion channel (VDAC) on the outer mitochondrial membrane and an adenine nucleotide translocase (ANT) on the inner mitochondrial membrane. However, the presence of Cr enhances this transfer by essentially concentrating ADP within the intermembrane space. The availability of phosphocreatine (PCr) essentially has the opposite effect by inhibiting the mitochondrial creatine kinase (miCK) reaction. It is currently estimated that ~80% of the energy transfer from matrix to cytosol occurs through miCK-dependent phosphate shuttling [32], although no direct evidence has been generated to support this supposition Modified slightly from Perry et al. [48]

The phosphate shuttling mechanism for energy transfer between matrix and cytosolic compartments includes three major protein complexes (see Fig. 2): voltage-dependent anion channel (VDAC) on the outer mitochondrial membrane, mitochondrial creatine kinase (miCK) in the inter membrane space, and adenine nucleotide translocase (ANT) on the inner mitochondrial membrane [32–34]. A leading model describing energy transfer from the matrix to cytosolic compartments proposes ATP produced within the matrix is transported to the inter membrane space via ANT where phosphate transfer to creatine (Cr) occurs through miCK. The PCr product is then transported through VDAC to the cytosol for ADP/ATP cycling via creatine kinase associated with cytosolic ATPases. While VDAC is involved in the transport of ADP into the mitochondria, VDAC has also been implicated in the transport of calcium and other metabolites into mitochondria [35, 36], as well as providing a docking site for cellular kinases [37], and in the regulation of apoptotic signaling [38–40]. Therefore, only miCK and ANT proteins are specific to ADP transport. While the current model proposes that ATP/ADP freely diffuse across concentration gradients through VDAC and ANT, it is estimated that as much as 80% of the energy transfer from the matrix to cytoplasm occurs through miCK-dependent phosphate shuttling in cardiac [32] and oxidative skeletal muscles [41]. Direct in vivo evidence implicating a prominent role for miCK has not been generated in skeletal muscle, as muscles lacking the miCK enzyme do not display compromised force production, altered fibre type, or metabolite levels [42], suggesting a limited role of miCK in regulating basal metabolism. Compensatory adaptions that offset the deleterious consequences of ablating miCK may exist, as it has been postulated that the cytosolic CK isoform redistributes to the outer mitochondrial membrane, although evidence for this supposition does not exist [43]. Alternately, Cr-independent regulation of mitochondrial ADP transport may be important. In support of this, we have recently shown that miCK knockout mice maintain exercise performance through activation of Cr-independent mechanisms [44]. ANT is required for both Cr dependent and independent transport of ADP/ATP across the inner mitochondrial membrane, and historically ADP flux through ANT was attributed solely to substrate levels [32]. While the simplicity of this proposed working model is appealing, external regulation of ANT likely exists. Indeed, ANT has several potential regulatory mechanisms including acetylation of lysine 23 [45], tyrosine 194 phosphorylation [46], and glutathionylation/carbonylation [47, 48]. While the functional roles for these regulatory points remain unknown, an acute bout of high-intensity interval exercise has been shown to decrease lysine 23 acetylation [45], a response predicted to improve the sensitivity to ADP binding. In support of this, in vitro assessments of mitochondrial ADP sensitivity indicate an improvement in ADP respiratory sensitivity in the absence of Cr after high-intensity exercise [49]. In contrast to these reports, steady-state exercise (~60% maximal rate of oxygen consumption for 2 h) attenuates mitochondrial ADP sensitivity in the absence of Cr [50], suggesting the regulation of ANT is highly complex and exercise intensity dependent. While our understanding of the regulation of mitochondrial ADP sensitivity is incomplete, palmitoyl-CoA is known to interact with ANT to inhibit the ADP/ATP exchange [51], a process attenuated by high-intensity exercise training [8]. High-intensity exercise training has also been shown to improve the sensitivity of Cr/PCr to stimulate/repress mitochondrial respiration, suggesting miCK is also influenced by chronic training [50]. Clearly, evidence is mounting to suggest that mitochondrial ADP sensitivity is regulated extensively during acute exercise and is influenced by chronic training. Combined, these data highlight the possibility of altering mitochondrial ADP sensitivity in the absence of mitochondrial biogenesis, challenging the long-held dogma that increases in mitochondrial content are required for fuel shifts and performance improvements.

## Nutritional Approaches to Augment Mitochondrial Bioenergetics

Traditional dogma stipulates that mitochondrial function is not externally regulated beyond the provision of substrates required for oxidative phosphorylation. This belief extends from Holloszy’s original observation that in vitro assessments of mitochondrial stoichiometry (P/O ratios: ADP consumed per oxygen atom) remain constant following chronic training [2]. However, it is now known that uncoupling proteins are externally regulated (e.g., glutathionylation of uncoupling protein 3 [52]), and evidence exists to show that the electron transport chain and ATP synthase are externally regulated [50, 53–55]. Clearly, the mitochondrion is a dynamic organelle, and the notion that lifestyle interventions cannot alter mitochondrial ‘efficiency’ needs to be reconsidered. This is best highlighted by the seminal finding that 3 days of dietary sodium nitrate improved mitochondrial coupling efficiency, increased maximal rates of ATP production, and reduced whole body oxygen consumption in humans [56, 57]. Intriguingly, the oral consumption of beetroot juice also decreases the oxygen cost of submaximal exercise in humans [58, 59], suggesting that oral nitrate sources universally improve mitochondrial respiratory efficiency. However, in contrast to sodium nitrate, beetroot juice does not alter isolated mitochondria P/O ratios [60], nor does it affect PCr resynthesis rates in vivo [61], suggesting the mechanism of action of beetroot juice does not involve an improvement in rates of ATP re-synthesis. Moreover, the provision of sodium nitrate reduced ANT protein content [57], and while this may improve mitochondrial coupling ratios, this would be expected to decrease mitochondrial ADP sensitivity, which would be counterproductive to training responses. Importantly, beetroot juice consumption does not alter mitochondrial ADP respiratory sensitivity or ANT protein content [60]. However, beetroot juice does increase mitochondrial ROS emission rates [60], which may contribute to the apparent improvement in excitation-contraction coupling efficiency following beetroot juice consumption [61, 62]. While currently speculative, pharmacological ablation of mitochondrial ROS (provision of SS31) attenuates low-frequency force production in rodent muscle [63], while in stark contrast, beetroot juice consumption increases mitochondrial ROS emission [59] and low-frequency stimulation force production in humans [62]. While a direct cause-and-effect relationship between mitochondrial ROS and an improvement in the mechanical efficiency of muscle remains to be determined, mechanisms pertaining to redox modifications within sarcomeres (e.g., troponin I) and calcium handling (e.g., RyR and the sarcoendoplasmic reticulum calcium ATPase) remain likely targets [64]. The necessity of mitochondrial ROS in mediating beetroot juice improvements in exercise performance is an attractive model given the known reduction in mitochondrial ROS following training [27], and the apparent resistance/attenuated response to beetroot juice in elite athletes [65–67]. It is also tempting to speculate that consumption of beetroot juice during a chronic training program would increase mitochondrial biogenesis as a result of an increase in mitochondrial ROS-mediated gene transcription; however, this possibility awaits direct scientific support, and it remains possible that the apparent increase in mitochondrial ROS following beetroot juice could be detrimental in some instances. Regardless, altogether the present data suggest that unlike sodium nitrate [55], the consumption of beetroot juice does not alter mitochondrial coupling efficiency in muscle [60, 61].

Given the relative novelty of identifying mitochondrial ADP transport as a regulated process, very little evidence has been generated with respect to nutritional approaches to augment this process. However, eicosapentaenoic acid (EPA) and docosahexaenoic acid (DHA) supplementation has been shown to alter the lipid composition of mitochondrial membranes in association with an increase in mitochondrial ADP sensitivity [68]. Intriguingly, EPA/DHA feeding also increases mitochondrial ROS emission rates [68], similar to beetroot juice consumption [60], raising the potential for this nutritional approach to improve exercise responses. In addition, EPA/DHA supplementation has been linked to improvements in protein synthesis, cognitive performance, immune function, bone integrity, and cardiovascular function and has been shown to increase the expression of genes associated with lipid oxidation in several tissues (reviewed by Jeromson et al. [69]). While it is therefore tempting to speculate that EPA/DHA supplementation can improve exercise performance, there is a paucity of literature surrounding the ability of EPA/DHA to improve metabolic responses during exercise in human skeletal muscle. Nevertheless, given the ability of EPA/DHA supplementation to improve mitochondrial ADP sensitivity, and the potential to induce mitochondrial biogenesis, research is clearly warranted to elucidate the ability of polyunsaturated fatty acid supplementation to augment exercise-induced adaptations and exercise performance. The interaction between polyunsaturated fatty acid nutritional interventions and exercise must be determined before advocating for athletes to incorporate this approach into a training regime, as there could be deleterious interactions, as exemplified by other nutritional interventions (e.g., resveratrol, see Gliemann et al. [70]).

## Conclusions

One of the most characterized responses to exercise training is an improvement in ADP sensitivity, sparing muscle glycogen and improving exercise capacity. Historically, these responses were exclusively attributed to an increase in mitochondrial content. However, recent evidence has accumulated to show that mitochondrial ADP transport is a highly regulated process. A better understanding of the molecular signals influencing the proteins directly controlling mitochondrial ADP transport, namely miCK and ANT, is required, as this knowledge could result in novel training programs aimed at optimizing muscle performance. While beetroot juice appears to influence excitation–contraction coupling, as opposed to mitochondrial bioenergetics, a key event in the observed improvement in exercise capacity may involve the increase in mitochondrial ROS. In addition, given the link between mitochondrial ROS and mitochondrial biogenesis, consumption of beetroot juice while training may induce mitochondrial biogenesis and indirectly improve ADP sensitivity. EPA/DHA supplementation similarly increases mitochondrial ROS emission rates, and because PUFAs are potent ligand activators of PPARs, incorporating PUFAs into a training program may augment chronic adaptations. This is particularly interesting, as EPA/DHA supplementation directly improves mitochondrial ADP sensitivity. Clearly, compelling evidence for a nutritional intervention that improves mitochondrial bioenergetics beyond the typical exercise-mediated responses does not exist. The recent advancement in our understanding of the regulation of mitochondrial ADP transport has elucidated gaps in our working models that need to be addressed. However, the awareness of these knowledge gaps creates the possibility for unique experimental approaches to be designed with the aim to improve exercise performance in the future.
